# Diphtheria

**Published:** 1876-04

**Authors:** 


Diphtheria—*By J. Solis Cohen, M.D.*—The paralyses that occur during convalescence, or after its establishment, or after the height of the disease has been passed, are indicative of a profound morbid impression on the nervous system. This impression is in some respects characteristic, although similar paralyses are found to occur after other severe specific systemic affections, as small-pox and typhoid fever. The amount and intensity of these paralyses of diphtheria are by no means directly proportionate to the severity of the affection.

The palate is the most frequent, and usually the earliest seat of the paralysis. It is seen to be relaxed, and does not contract upon contact with it of instruments or stimulus. The results are difficulty of swallowing, regurgitation of fluids through the nose, and an altered (so miscalled nasal) tone of voice. It seldom occurs before the third or fourth week of the disease, although it has been noted as early as the ninth day. Cutaneous diphtheria is sometimes followed by the same sort of paralysis of the palate, a significant fact indicating that the impairment of nervous force is due to systemic poisoning rather than to any local effect upon the peripheral terminations of the affected nerves themselves.

The pharynx, and, to some extent, the œsophagus also, are sometimes paralytic after diphtheria; under which circumstances the difficulty of swallowing is still greater, so great sometimes as to necessitate feeding through a stomach tube. Vomiting, however, may occur even after swallowing is impossible. The nerves of the larynx may be paralyzed, in which case there will be impairment of voice, constriction of the glottis, or immobility of the epiglottis, according to the nerves impaired. Laryngeal paralysis, favoring the escape of food into the air-passages, necessitates the use of the stomach tube. Constriction of the glottis would be an indication for tracheotomy. The nerves of respiration are sometimes impaired, and we have dyspnœa in consequence, so that the institution of artificial respiration may be temporarily requisite from time to time. The cardiac nerves may become implicated, a manifestation, often, of fatal significance, the activity of the heart diminishing to from sixty to forty pulsations in the minute, or down to thirty, and even below that before death.

In addition to these manifestations there may be impairment of the organs of special sense : taste, smell, hearing, and vision. In fact, the paralyses may be manifested in any of the organs and muscles in which paralysis is manifested under other conditions.

The treatment of the paralysis following diphtheria consists in the use of the ordinary methods of invigoration—fresh air, baths, and tonics—under the influence of which the nervous system gradually undergoes recuperation, and the paralyses subside. Should this not be the case, the use of strychnia and of phosphorus are indicated, while local applications of the electric current are calculated to hasten the progress of restoration to normal function. Symptoms of paralysis sometimes remain for several months after convalescence from the diphtheric manifestations. Occasionally they are insusceptible of amelioration, and if important functions are interfered with they may be the direct cause of death, as in paralysis of the respiratory organ, the heart, and the pharynx and œsophagus.

